# Visceral fat area and visceral-to-subcutaneous fat ratio are more strongly associated with residual cholesterol than conventional anthropometric indices in adults with type 2 diabetes

**DOI:** 10.3389/fendo.2026.1848101

**Published:** 2026-05-29

**Authors:** Zichen Rao, Liangyan Hua, Yiwei Mo, Zhenghao Wu, Ziru Fang, Chunyan Zhu, Lifang Cheng, Yiming Zhang

**Affiliations:** 1 Department of Endocrinology, The Quzhou Affiliated Hospital of Wenzhou Medical University, Quzhou People’s Hospital, Quzhou, Zhejiang, China; 2 Department of General Practice, Chihuai Branch, Kaihua County People’s Hospital, Quzhou, Zhejiang, China

**Keywords:** anthropometric indices, cardiometabolic risk, central obesity, residual cholesterol, type 2 diabetes, visceral fat area, visceral-to-subcutaneous fat ratio

## Abstract

**Background:**

Residual cholesterol (RC) is linked to cardiovascular risk, yet the optimal adiposity indices to capture this burden in type 2 diabetes (T2D) remain unclear. We compared associations of general and central adiposity indices with RC in a real-world T2D cohort.

**Methods:**

This cross-sectional study included 1,135 adults with T2D at a Chinese Metabolic Management Centre (2022–2025). We assessed BMI, weight, waist circumference (WC), waist-to-height ratio (WHtR), visceral fat area (VFA), subcutaneous fat area (SFA), and VFA/SFA ratio. Linear regression estimated β coefficients for RC per 1-SD increase in indices, adjusted for age, sex, HbA1c, eGFR, blood pressure, smoking, and drinking. Nonlinearity was assessed using generalized additive models and piecewise regression.

**Results:**

Across RC quartiles, patients with higher RC were more often male and current smokers and had higher BMI, WC, VFA, SFA, WHtR and VFA/SFA. In unadjusted models, all adiposity indices were positively associated with RC, but after full adjustment only VFA (β 0.1102, 95% CI 0.0186–0.2018) and VFA/SFA (β 0.1882, 95% CI 0.1010–0.2753) remained independently related to RC. When modelled in original units and quartiles, the highest VFA quartile was associated with 0.383 mmol/L (95% CI 0.131–0.636) higher RC and the highest VFA/SFA quartile with 0.562 mmol/L (95% CI 0.316–0.808) higher RC compared with the lowest quartile in fully adjusted models. A non-linear association between VFA/SFA and RC was observed, with an inflection around 0.72: below this threshold, higher VFA/SFA was strongly associated with higher RC (β 1.61, 95% CI 0.91–2.30), whereas no clear association was seen above it. Subgroup analyses showed broadly consistent associations across age, sex, BMI and HbA1c strata, without significant interactions.

**Conclusions:**

In T2D, indices of visceral fat accumulation—particularly VFA and VFA/SFA—are more strongly associated with RC than BMI, WC, or WHtR. A VFA/SFA ratio near 0.7–0.8 may mark a critical range where visceral fat predominance is most strongly linked to RC elevation. Detailed visceral adiposity assessment may help refine RC-related risk stratification beyond traditional measures.

## 1 Introduction

Type 2 diabetes (T2D) is a major global health problem strongly associated with an excess risk of cardiovascular disease (CVD) (1, 2). Even when low-density lipoprotein cholesterol (LDL-C) is lowered to targets, many individuals with T2D continue to experience cardiovascular events, a phenomenon widely referred to as residual cardiovascular risk (3–5). Residual cholesterol (RC), representing the cholesterol content of triglyceride-rich remnants, has emerged as a simple and robust marker of this risk (6). Higher RC levels are independently associated with incident CVD and mortality, particularly in East Asian and diabetic populations who frequently present with hypertriglyceridaemia and small dense LDL particles (7–9). However, in real-world T2D cohorts, the determinants of elevated RC remain incompletely understood, and it is still unclear which easily obtainable clinical indices best reflect RC burden in daily practice (10).

Obesity is a key upstream driver of cardiometabolic complications and dyslipidaemia in T2D (11, 12). Although traditional anthropometric indices such as body weight, body mass index (BMI), and waist circumference (WC) are widely used to characterize obesity, they cannot distinguish between visceral and subcutaneous fat, providing limited information on ectopic fat deposition (13, 14). Imaging and body composition studies have demonstrated that individuals with identical BMI or WC can exhibit markedly different amounts of visceral adipose tissue and divergent cardiometabolic risks (15). Visceral fat deposition shows a much stronger association with insulin resistance and triglyceride-rich lipoprotein accumulation than overall adiposity (16). Consequently, relying solely on traditional measures may misclassify metabolically vulnerable individuals, prompting a growing interest in obesity indices that more precisely capture visceral adiposity as a potential driver of RC elevation in T2D (17–19).

With advances in body composition assessment, indices reflecting abdominal fat distribution have become clinically accessible (20). Visceral fat area (VFA) and subcutaneous fat area (SFA), increasingly estimated via practical methods like bioelectrical impedance analysis, provide direct quantification of abdominal fat compartments (21). The visceral-to-subcutaneous fat ratio (VFA/SFA) has been proposed to indicate visceral predominance and is linked to higher cardiovascular risk (22), while central adiposity indices like the waist-to-height ratio (WHtR) and visceral adiposity index have shown unique performance in screening cardiometabolic risks in Asian populations (23, 24). However, evidence directly comparing a broad panel of general and central adiposity indices—including body weight, BMI, WC, WHtR, VFA, SFA, and the VFA/SFA ratio—in relation to RC remains limited, particularly in adults with T2D (25).

Against this background, we conducted a cross-sectional study in adults with T2D enrolled in a single Metabolic Management Centre to systematically compare the associations of these general and central adiposity indices with RC (26, 27). We additionally explored potential non-linear relationships and threshold effects for key indices, and evaluated their consistency across various clinical subgroups (28). We hypothesized that indices reflecting visceral adiposity and its predominance, particularly VFA and the VFA/SFA ratio, would demonstrate stronger independent associations with RC than traditional anthropometric measures, thereby refining RC-related risk stratification in routine clinical practice (29).

## 2 Materials

### 2.1 Study population

This cross-sectional study was conducted at the Metabolic Management Centre (MMC) of the Department of Endocrinology, Quzhou People’s Hospital, an affiliated hospital of Wenzhou Medical University in Quzhou, Zhejiang, China. We included consecutive adult patients with type 2 diabetes (T2D) who underwent standardized baseline evaluation at the MMC between December 2022 and June 2025, using de-identified data extracted retrospectively from routine clinical records. T2D was defined according to the 2023 American Diabetes Association (ADA) criteria.

Patients were eligible if they met all of the following criteria: (i) age ≥18 years; (ii) a confirmed diagnosis of T2D; and (iii) completion of the MMC baseline assessment with available measurements of body weight, height, waist circumference (WC), VFA, subcutaneous fat area (SFA), and a fasting lipid profile.

**Figure 1:**
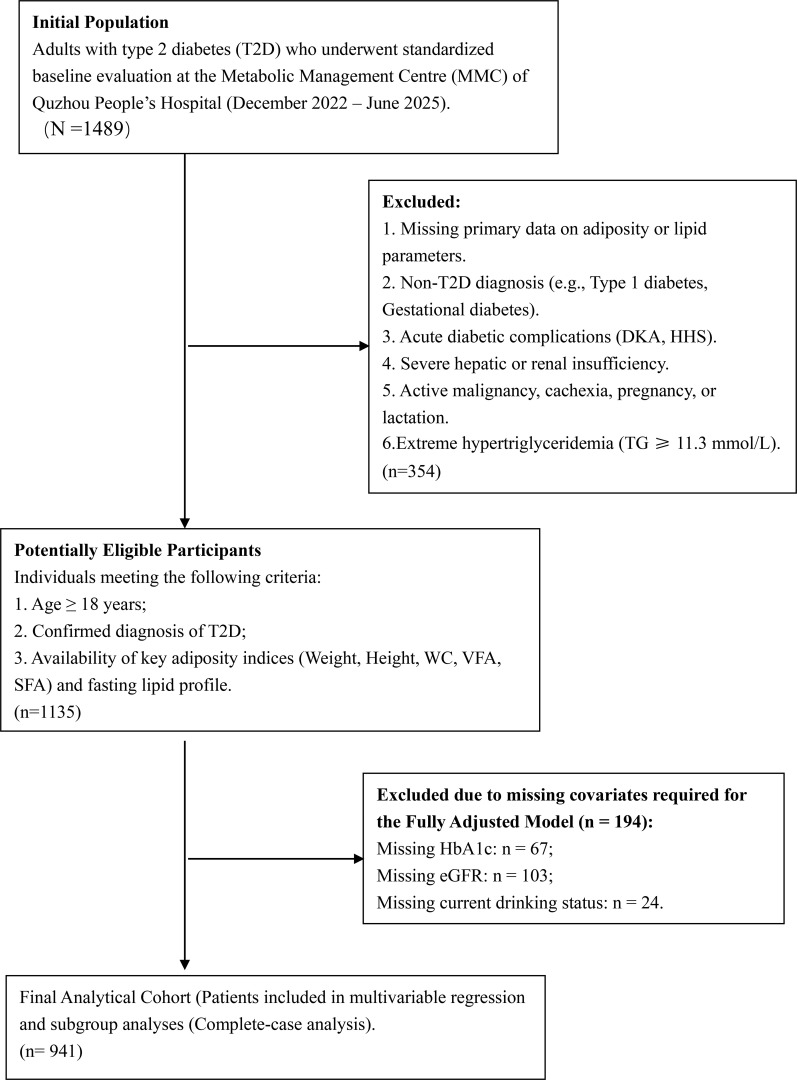
Flowchart of participant selection.

We excluded patients with acute diabetic complications at the time of evaluation (e.g. diabetic ketoacidosis or hyperosmolar hyperglycaemic crisis), type 1 diabetes, gestational diabetes or other specific types of diabetes. Additional exclusion criteria were known severe hepatic or renal insufficiency (such as decompensated cirrhosis, end-stage renal disease or maintenance dialysis), clinically evident acute infection or other acute inflammatory diseases, active malignancy or cachexia, pregnancy or lactation, missing data on key adiposity indices, or lipids parameters, and triglyceride (TG) levels ≥11.3 mmol/L, in order to minimize the influence of extreme hypertriglyceridaemia on cholesterol measures. After applying these criteria, 1,135 adults with T2D were included in the descriptive analyses. Among them, 941 patients had complete data on all covariates and were included in the multivariable and subgroup analyses. The detailed participant selection process is illustrated in **Figure 1**.

### 2.2 Clinical and laboratory measurements

Trained nurses collected baseline demographic and clinical data, including age, sex, smoking and drinking status, and medical history, through routine clinical interviews during the initial MMC assessment.

Body weight and height were measured with participants wearing light clothing and no shoes, and BMI was calculated as weight in kilograms divided by height in meters squared (kg/m²). WC was measured to the nearest 0.1cm at the midpoint between the lowest rib and the iliac crest after a normal expiration, using a non-elastic tape. In the MMC, VFA and SFA were assessed with a multifrequency bioelectrical impedance body composition analyzer, according to the manufacturer’s instructions and standardized operating procedures.

Blood pressure was measured on the right arm in the seated position after at least 5 minutes of rest, using an automated sphygmomanometer. Two measurements were obtained 1–2 minutes apart, and the average values of systolic blood pressure (SBP) and diastolic blood pressure (DBP) were used in the analyses.

Fasting venous blood samples were collected in the morning after an overnight fast of at least 8 hours. Glycated hemoglobin (HbA1c) was determined by high-performance liquid chromatography. Serum lipids, including total cholesterol (TC), TG, HDL-C and LDL-C, as well as serum creatinine, uric acid, alanine aminotransferase (ALT) and aspartate aminotransferase (AST), were measured on automated analyzers using standard enzymatic methods under routine internal and external quality control. Estimated glomerular filtration rate (eGFR) was calculated from serum creatinine, age and sex using the CKD-EPI equation.

Definitions of adiposity indices and residual cholesterol.

In this study, we focused on several conventional and central adiposity indices. BMI was calculated as described above. Waist-to-height ratio (WHtR) was derived as WC (cm) divided by height (cm). VFA and SFA were obtained directly from the bioelectrical impedance analyzer. Body weight, WC, WHtR, VFA, SFA and the VFA/SFA ratio were treated as continuous variables in the main analyses, and alternative categorizations (e.g. quartiles) were used to explore dose–response patterns.

Residual cholesterol (RC) was calculated from the standard fasting lipid profile using the following formula:

RC=TC−LDL-C−HDL-C.

All lipid parameters, including RC, were expressed in mmol/L.

### 2.3 Ethical considerations

The study was conducted in accordance with the Declaration of Helsinki. The protocol was reviewed and approved by the Ethics Committee of Quzhou People’s Hospital, Quzhou Affiliated Hospital of Wenzhou Medical University (Approval No. 2022–110). The participants provided their written informed consent to participate in this study.

### 2.4 Statistical analysis

Baseline characteristics across RC quartiles were described as median (interquartile range) for continuous variables and as numbers (percentages) for categorical variables. Continuous variables were inspected graphically and were mostly non-normally distributed; therefore, group differences were assessed using the Kruskal–Wallis test for continuous variables and the χ² test for categorical variables.

The initial study population comprised 1,135 eligible participants. For multivariable analyses, a complete-case approach was adopted, resulting in an analytical sample of 941 participants after excluding those with missing covariates. *Post-hoc* power calculations using G*Power software (version 3.1.9.7) indicated that a sample of 941 provides over 99% power to detect a correlation coefficient of 0.15 at a two-sided alpha level of 0.05, confirming the adequacy of the sample size for the reported associations.

To examine the association between adiposity indices and RC, linear regression models were fitted with RC (mmol/L) as the dependent variable. BMI, VFA, SFA, body weight, WC, WHtR and the VFA/SFA ratio were standardized to Z-scores, and β coefficients with 95% confidence intervals (CIs) were reported per 1-standard deviation increase. Three models were specified: Model 0 was unadjusted; Model 1 was adjusted for age and sex; and Model 2 was further adjusted for HbA1c, eGFR, systolic blood pressure, current smoking and current drinking. For VFA, WC, WHtR and the VFA/SFA ratio, additional models treated these variables in their original units and as quartiles (reference: Q1) to characterize dose–response patterns. The effective sample size for each multivariable and subgroup analysis is shown in the tables.

Non-linear associations between the VFA/SFA ratio and RC were assessed using a generalized additive model with a smoothing spline based on the covariates in Model 2. When non-linearity was suggested, a two-piecewise linear regression model was applied to estimate a data-driven inflection point and the slopes below and above this point, and a likelihood ratio test was used to compare the two-piecewise model with a single-line model. Subgroup analyses were performed for age (<60 vs ≥60 years), sex (men vs women), BMI (<28 vs ≥28 kg/m²) and HbA1c (<7% vs ≥7%) using fully adjusted models, and P values for interaction were obtained from product terms between the VFA/SFA ratio and the stratifying variables. The age threshold was selected to evaluate physiological variations associated with biological senescence and age-related visceral fat redistribution. The cut-off values for BMI and HbA1c were predefined based on the localized criteria for adult obesity by the National Health Commission of China (WS/T 428-2013) and the standard clinical target for optimal glycaemic control, respectively. All tests were two-sided, and P<0.05 was considered statistically significant. Analyses were conducted using EmpowerStats (X&Y Solutions Inc., Boston, MA, USA) and R (R Foundation for Statistical Computing, Vienna, Austria).

## 3 Results

### 3.1 Baseline characteristics by residual cholesterol quartiles

A total of 1,135 adults with type 2 diabetes were divided into quartiles of residual cholesterol (RC, Q1–Q4; **Table 1**). From the lowest to the highest quartile, patients tended to be younger, while the proportions of men, current smokers and higher diastolic blood pressure gradually increased; systolic blood pressure was broadly similar across groups. HbA1c levels were high in all quartiles without a clear monotonic trend, and patients with higher RC had slightly higher eGFR, uric acid, creatinine and ALT. Regarding lipids, total cholesterol and triglycerides increased across RC quartiles, HDL-C decreased, and LDL-C was paradoxically lower in the higher RC groups. Notably, indices of overall and central adiposity showed clear gradients: waist circumference, body weight, BMI, waist-to-height ratio, visceral fat area and subcutaneous fat area all rose from Q1 to Q4, and the VFA/SFA ratio also increased (median 0.53 in Q1 vs 0.59 in Q4).

**Table 1:** Baseline characteristics of adults with type 2 diabetes according to quartiles of residual cholesterol.

Characteristic	Q1	Q2	Q3	Q4	*P*-value
N	281	283	280	291	
AGE (years)	56.00 (48.00-63.00)	57.00 (50.00-65.00)	55.00 (47.00-62.25)	49.00 (39.50-59.00)	<0.001
SEX (%)					0.037
Female	112 (39.86%)	104 (36.75%)	108 (38.57%)	85 (29.21%)	
Male	169 (60.14%)	179 (63.25%)	172 (61.43%)	206 (70.79%)	
Current drinking, n (%)					0.136
No	183 (70.38%)	185 (70.34%)	173 (67.05%)	166 (59.71%)	
Yes	77 (29.62%)	78 (29.66%)	85 (32.95%)	112 (40.29%)	
Current smoking, n (%)					<0.001
No	188 (72.03%)	187 (71.10%)	181 (70.16%)	153 (55.04%)	
Yes	73 (27.97%)	76 (28.89%)	77 (29.85%)	125 (44.96%)	
DBP (mmHg)	74.00 (69.00-78.00)	75.00 (70.00-79.00)	75.00 (70.00-79.25)	76.00 (72.00-83.00)	<0.001
SBP (mmHg)	126.00 (117.00-134.00)	126.00 (119.50-136.00)	126.00 (118.00-136.00)	126.00 (120.00-137.50)	0.354
HbA1C (%)	9.17 (7.25-10.99)	8.80 (7.29-10.55)	8.91 (7.20-10.58)	9.63 (7.85-11.25)	0.092
TC (mmol/L)	4.56 (3.73-5.36)	4.29 (3.62-4.97)	4.48 (3.86-5.16)	5.13 (4.47-6.04)	<0.001
HDLC (mmol/L)	1.17 (0.98-1.33)	1.15 (0.98-1.33)	1.06 (0.90-1.29)	1.01 (0.85-1.18)	<0.001
LDL-C (mmol/L)	3.14 (2.38-3.78)	2.67 (2.00-3.40)	2.63 (2.01-3.34)	2.51 (1.89-3.15)	<0.001
TG (mmol/L)	1.13 (0.90-1.42)	1.48 (1.15-1.92)	2.27 (1.73-2.95)	4.62 (3.17-6.03)	<0.001
EGFR (mL/min/1.73 m²)	103.71 (95.84-113.13)	102.16 (92.94-110.74)	100.91 (91.80-110.66)	107.71 (94.93-117.20)	<0.001
SCr	59.70 (51.60-70.00)	63.05 (52.62-72.55)	65.80 (55.20-77.10)	64.20 (55.30-76.40)	<0.001
UA (umol/L)	297.30 (248.57-354.50)	298.00 (245.28-361.57)	322.75 (270.98-379.50)	351.10 (291.80-415.30)	<0.001
AST (U/L)	18.00 (14.45-25.85)	17.80 (15.00-24.50)	19.35 (15.70-25.85)	19.85 (15.10-26.65)	0.242
ALT (U/L)	21.20 (13.75-37.00)	19.60 (14.10-34.30)	23.00 (16.30-35.75)	25.70 (17.20-41.70)	<0.001
CRP (mg/L)	1.40 (0.81-3.20)	1.35 (0.55-4.03)	1.60 (0.73-3.43)	1.83 (0.92-3.57)	0.101
WC (cm)	86.00 (81.38-93.00)	88.00 (83.00-94.00)	89.50 (84.00-96.68)	91.80 (86.10-97.75)	<0.001
VFA (cm^2^)	87.00 (66.00-108.00)	87.00 (67.00-115.00)	94.00 (71.50-119.00)	107.50 (80.75-126.25)	<0.001
SFA (cm^2^)	156.00 (122.00-201.00)	163.00 (126.00-214.00)	168.00 (133.00-212.00)	181.50 (144.75-220.75)	0.001
WEIGHT	64.40 (58.40-71.60)	66.00 (59.65-73.65)	67.25 (59.92-76.93)	72.10 (64.10-80.00)	<0.001
HEIGHT	162.50 (156.00-168.50)	165.00 (157.50-170.00)	165.00 (157.00-170.00)	167.50 (160.25-172.00)	<0.001
WHtR (Waist-to-Height Ratio)	0.53 (0.49-0.57)	0.54 (0.50-0.57)	0.55 (0.52-0.58)	0.55 (0.51-0.59)	<0.001
BMI (kg/m²)	24.50 (22.30-26.90)	24.50 (22.55-26.70)	25.15 (23.08-27.90)	26.00 (24.00-27.90)	<0.001
VFA/SFA	0.53 (0.42-0.65)	0.55 (0.43-0.65)	0.55 (0.46-0.65)	0.59 (0.50-0.67)	0.002

Data are presented as median (interquartile range) or n (%), as appropriate. Because most continuous variables were skewed, non-parametric tests were used. P values refer to comparisons across residual cholesterol (RC) quartiles using the Kruskal–Wallis test for continuous variables and the χ² test for categorical variables.

BMI, body mass index; WC, waist circumference; WHtR, waist-to-height ratio; VFA, visceral fat area; SFA, subcutaneous fat area; SBP, systolic blood pressure; DBP, diastolic blood pressure; eGFR, estimated glomerular filtration rate; ALT, alanine aminotransferase; AST, aspartate aminotransferase; CRP, C-reactive protein; TC, total cholesterol; TG, triglycerides; HDL-C, high-density lipoprotein cholesterol; LDL-C, low-density lipoprotein cholesterol. SCr, serum creatinine; UA, uric acid.

### 3.2 Associations of adiposity indices with residual cholesterol

In linear regression analyses with RC as the outcome, all adiposity indices were positively associated with RC in the unadjusted models. Each 1-SD increase in the Z-scores for BMI, VFA, subcutaneous fat area (SFA), body weight, waist circumference (WC), waist-to-height ratio (WHtR) and the VFA/SFA ratio corresponded to approximately 0.09–0.17 mmol/L higher RC (all P<0.01; **Table 2**). These associations were only modestly attenuated after adjustment for age and sex. However, in the fully adjusted model, which additionally included HbA1c, eGFR, systolic blood pressure, current smoking and current drinking, most adiposity indices were no longer significantly related to RC. For example, the β coefficient for BMI Z-score decreased to 0.0002 (95% CI −0.0826 to 0.0829; P=0.996), and the estimates for body weight, WC, WHtR and SFA Z-scores also approached the null. By contrast, VFA and the VFA/SFA ratio remained independently associated with RC, with β 0.1102 (95% CI 0.0186 to 0.2018; P=0.018) for VFA Z-score and β 0.1882 (95% CI 0.1010 to 0.2753; P<0.001) for VFA/SFA Z-score in Model 2, the latter showing the largest effect size among all adiposity indices, which prompted more detailed analyses of these measures in subsequent models.

**Table 2:** Associations of adiposity indices (per 1-SD increase) with residual cholesterol in adults with type 2 diabetes.

Exposure	Crude model (Model 0) β (95% CI), *p*-value	Partially adjusted model (Model 1) β (95% CI), *p*-value	Fully adjusted model (Model 2) β (95% CI), *p*-value
BMI Z-score	0.1226 (0.0658, 0.1794) <0.001	0.0772 (0.0191, 0.1352) 0.009	0.0002 (-0.0826, 0.0829) 0.996
VFA Z-score	0.1709 (0.1071, 0.2347) <0.001	0.1498 (0.0862, 0.2134) <0.001	0.1102 (0.0186, 0.2018) 0.018
SFA Z-score	0.0970 (0.0304, 0.1637) 0.004	0.0652 (-0.0018, 0.1322) 0.057	-0.0337 (-0.1316, 0.0642) 0.500
WEIGHT Z-score	0.1617 (0.1068, 0.2166) <0.001	0.0973 (0.0324, 0.1622) 0.003	0.0253 (-0.0657, 0.1163) 0.586
WC Z-score	0.1498 (0.0939, 0.2056) <0.001	0.1241 (0.0683, 0.1800) <0.001	0.0623 (-0.0152, 0.1398) 0.115
WHtR Z-score	0.0898 (0.0335, 0.1462) 0.002	0.1123 (0.0560, 0.1685) <0.001	0.0468 (-0.0312, 0.1248) 0.240
VFA/SFA Z-score	0.1174 (0.0529, 0.1820) <0.001	0.1344 (0.0688, 0.1999) <0.001	0.1882 (0.1010, 0.2753) <0.001

Analyses were restricted to participants with complete data on all covariates (n=941).

Outcome variable: RC (mmol/L). Values are β (95% CI) and P value per 1-standard deviation increase in each adiposity index from linear regression models.

Model 0, unadjusted.

Model 1, adjusted for age and sex.

Model 2, further adjusted for HbA1c, eGFR, SBP, current smoking and current drinking.

Abbreviations as in **Table 1**.

### 3.3 Associations of key adiposity measures with RC

When VFA, WC, WHtR and the VFA/SFA ratio were modelled as continuous variables, all four indices showed positive associations with RC in the unadjusted and age- and sex-adjusted models (**Table 3**). In the fully adjusted model, the associations for WC and WHtR were attenuated and no longer significant, whereas higher VFA and a higher VFA/SFA ratio remained independently related to higher RC (β 0.003, 95% CI 0.000–0.005; P=0.019 for VFA; β 1.111, 95% CI 0.596–1.625; P<0.001 for VFA/SFA). Quartile-based analyses yielded similar patterns: compared with the lowest quartile, the highest VFA quartile was associated with 0.383 mmol/L (95% CI 0.131–0.636; P=0.003) higher RC, and the highest VFA/SFA quartile was associated with 0.562 mmol/L (95% CI 0.316–0.808; P<0.00001) higher RC after full adjustment.

**Table 3:** Associations of VFA, WC, WHtR and VFA/SFA with residual cholesterol in adults with type 2 diabetes.

Exposure	Crude model (Model 0) β (95% CI), *p*-value	Partially adjusted model (Model 1) β (95% CI), *p*-value	Fully adjusted model (Model 2) β (95% CI), *p*-value
RC (mmol/L)			
VFA	0.004 (0.003, 0.006) <0.00001	0.004 (0.002, 0.005) <0.00001	0.003 (0.000, 0.005) 0.01864
Q1	1.0	1.0	1.0
Q2	0.079 (-0.104, 0.262) 0.39580	0.077 (-0.103, 0.257) 0.40391	0.088 (-0.143, 0.318) 0.45641
Q3	0.280 (0.095, 0.466) 0.00311	0.264 (0.080, 0.448) 0.00498	0.319 (0.074, 0.565) 0.01114
Q4	0.424 (0.240, 0.609) <0.00001	0.355 (0.171, 0.539) 0.00016	0.339 (0.086, 0.593) 0.00897
P for trend	<0.00001	0.00003	0.00209
WC	0.015 (0.009, 0.021) <0.00001	0.012 (0.007, 0.018) 0.00001	0.006 (-0.002, 0.014) 0.11542
Q1	1.0	1.0	1.0
Q2	0.057 (-0.100,0.213) 0.47626	0.061 (-0.094, 0.216) 0.43971	0.078 (-0.116, 0.272) 0.43163
Q3	0.339 (0.183, 0.496) 0.00002	0.322 (0.167, 0.477) 0.00005	0.293 (0.092, 0.493) 0.00435
Q4	0.337 (0.181, 0.493) 0.00003	0.279 (0.123, 0.434) 0.00045	0.167 (-0.044, 0.378) 0.12083
P for trend	<0.00001	0.00001	0.02825
WHtR	1.473 (0.548, 2.397) 0.00184	1.840 (0.918, 2.763) 0.00010	0.768 (-0.511, 2.046) 0.23983
Q1	1.0	1.0	1.0
Q2	0.103 (-0.055,0.261) 0.20018	0.111 (-0.044, 0.265) 0.16015	0.102 (-0.088, 0.293) 0.29377
Q3	0.132 (-0.025,0.290) 0.10072	0.165 (0.010, 0.319) 0.03687	0.161 (-0.043, 0.365) 0.12217
Q4	0.248 (0.090, 0.405) 0.00209	0.301 (0.144, 0.458) 0.00018	0.160 (-0.050, 0.369) 0.13492
P for trend	0.00240	0.00017	0.10677
VFA/SFA	0.693 (0.312, 1.074) 0.00038	0.793 (0.406, 1.180) 0.00006	1.111 (0.596, 1.625) 0.00003
Q1	1.0	1.0	1.0
Q2	0.141 (-0.042, 0.323) 0.13072	0.145 (-0.036, 0.325) 0.11677	0.198 (-0.040, 0.436) 0.10426
Q3	0.273 (0.090, 0.455) 0.00346	0.272 (0.088, 0.455) 0.00377	0.373 (0.130, 0.615) 0.00273
Q4	0.343 (0.161, 0.525) 0.00024	0.381 (0.195, 0.568) 0.00007	0.568 (0.324, 0.812) <0.00001
P for trend	0.00008	0.00003	<0.00001

Analyses were restricted to participants with complete data on all covariates (n=941).

Outcome variable: RC (mmol/L). Values are β (95% CI) and P value from linear regression models with each adiposity measure entered either as a continuous variable or as quartiles (reference: Q1).

Model 0: unadjusted.

Model 1: adjusted for age and sex.

Model 2: further adjusted for HbA1c, eGFR, systolic blood pressure, current smoking and current drinking.

P for trend was assessed by modeling the median value of each quartile as a continuous variable.

Abbreviations as in **Table 1**.

#### 3.4 Non-linear association between VFA/SFA and residual cholesterol

**Figure 2:**
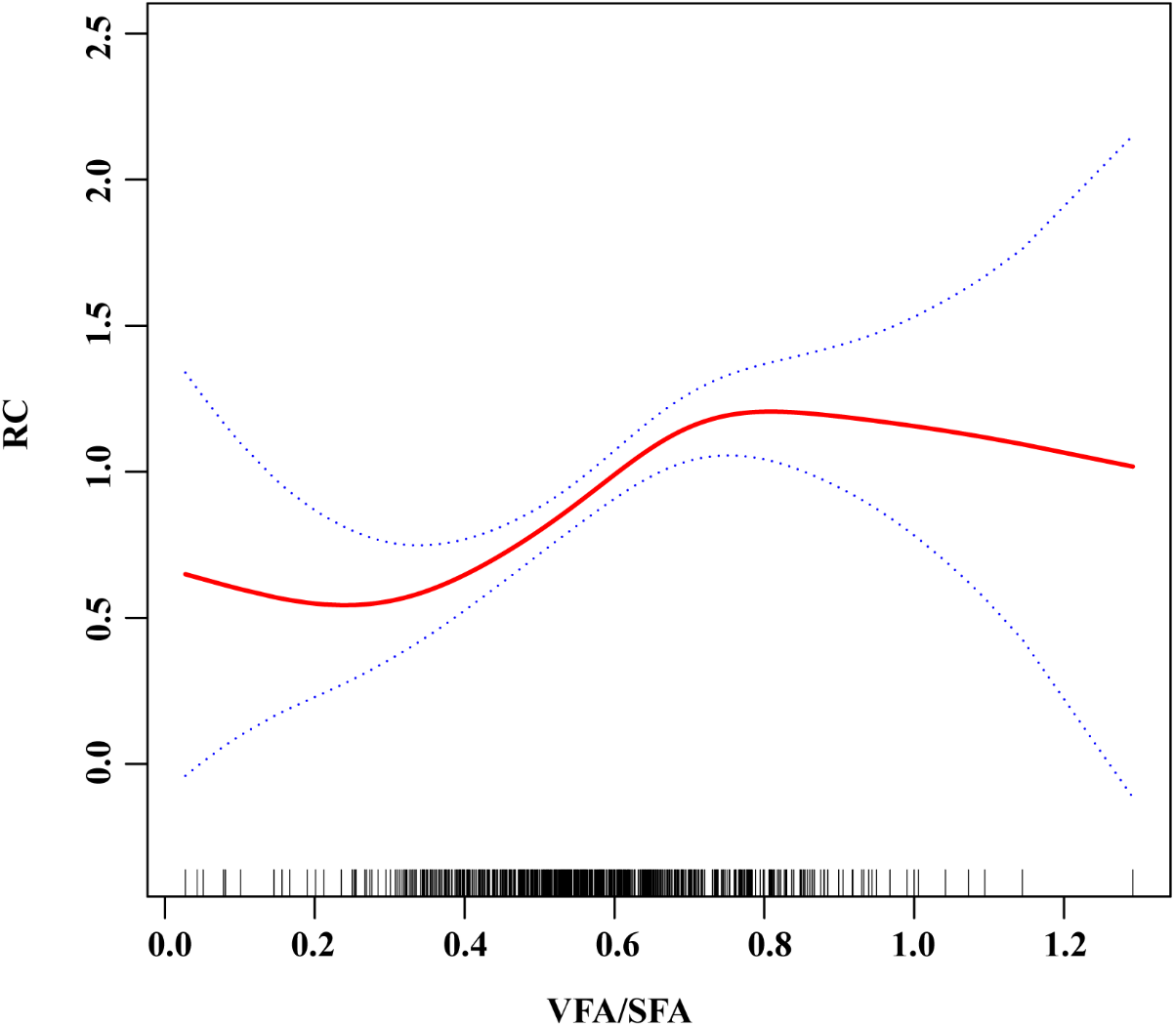
Adjusted smooth curve of the association between the VFA/SFA ratio and residual cholesterol in adults with type 2 diabetes.

To further characterize the relationship between fat distribution and RC, we examined the association between the VFA/SFA ratio and RC using a smoothing spline based on the fully adjusted model. The adjusted curve showed an approximately linear increase in RC with rising VFA/SFA up to around 0.7, followed by an attenuation of the positive association and a slight decline at higher values (**Figure 2**). In a subsequent threshold-effect analysis using a two-piecewise linear regression model, an inflection point was identified at a VFA/SFA ratio of 0.72 (**Table 4**). Below this threshold, higher VFA/SFA was strongly associated with higher RC (β 1.61, 95% CI 0.91–2.30; P<0.001), whereas above 0.72 the association was attenuated and no longer statistically significant (β −0.49, 95% CI −2.08–1.10; P=0.55). The two-segment model provided a significantly better fit than the single-line model (likelihood ratio test P=0.036), supporting the presence of a potential threshold around a VFA/SFA ratio of 0.7–0.8.

**Table 4:** Threshold effect analysis of the association between the VFA/SFA ratio and residual cholesterol.

RC	Adjusted β (95% CI)	*P*-value
VFA/SFA (Model I)	1.11 (0.60, 1.63)	<0.0001
VFA/SFA (Model II)		
Inflection point	0.72	
VFA/SFA <0.72	1.61 (0.91, 2.30)	<0.0001
VFA/SFA >=0.72	-0.49 (-2.08, 1.10)	0.5478
Difference (Segment 2 – 1)	-2.09 (-4.06, -0.12)	0.0376
Log likelihood ratio		0.036

Outcome variable: RC (mmol/L).

Estimates are derived from two-piecewise linear regression models with a data-driven inflection point for the visceral-to-subcutaneous fat area (VFA/SFA) ratio. Segment 1 represents the slope below the inflection point and segment 2 the slope above it. The model was adjusted for age, sex, HbA1c, eGFR, systolic blood pressure, current smoking and current drinking. Abbreviations as in **Table 1**.

The solid line represents the estimated association between the visceral-to-subcutaneous fat area (VFA/SFA) ratio and residual cholesterol (RC) from a generalized additive model, and the shaded area indicates the 95% confidence interval. The model was adjusted for age, sex, HbA1c, eGFR, systolic blood pressure, current smoking and current drinking.

### 3.5 Subgroup analyses stratified by age, sex, BMI and HbA1c

In subgroup analyses, the positive association between the VFA/SFA ratio and RC was generally consistent across strata of age, sex, BMI and HbA1c (**Table 5**). When stratified by age, higher VFA/SFA was associated with higher RC in both patients <60 years (β 0.996, 95% CI 0.235–1.757; P=0.011) and those ≥60 years (β 1.032, 95% CI 0.562–1.501; P<0.001), with no evidence of interaction (P for interaction=0.96). By sex, the association was stronger and statistically significant in men (β 1.159, 95% CI 0.515–1.802; P=0.001), while in women the estimate was weaker and did not reach conventional significance (β 0.805, 95% CI −0.064–1.674; P=0.071), although the interaction by sex was not significant (P for interaction=0.76). Stratification by BMI showed that VFA/SFA was clearly related to RC in patients with BMI <28 kg/m² (β 1.223, 95% CI 0.660–1.786; P<0.001), whereas no association was observed in those with BMI ≥28 kg/m² (β −0.052, 95% CI −1.340–1.236; P=0.94; P for interaction=0.17). Finally, the association appeared more pronounced in patients with HbA1c ≥7% (β 1.040, 95% CI 0.443–1.637; P=0.001) than in those with HbA1c <7% (β 0.540, 95% CI −0.206–1.286; P=0.16), but the interaction by HbA1c level was not statistically significant (P for interaction=0.50).

**Table 5:** Subgroup analyses of the association between the VFA/SFA ratio and residual cholesterol.

Subgroup	N	β (95% CI)	P-value	P for interaction
AGE				0.9625
<60	631	0.996 (0.235, 1.757)	0.0107	
≥60	310	1.032 (0.562, 1.501)	<0.0001	
Gender				0.7646
Female	339	0.805 (-0.064, 1.674)	0.0709	
Male	602	1.159 (0.515, 1.802)	0.0005	
BMI				0.1674
<28	722	1.223 (0.660, 1.786)	<0.0001	
≥28	219	-0.052 (-1.340, 1.236)	0.9371	
HbA1c				0.5029
<7%	270	0.540 (-0.206, 1.286)	0.1596	
≥7%	559	1.040 (0.443, 1.637)	0.0007	

Outcome variable: RC (mmol/L).

Values are β (95% CI) and P value for the association between the visceral-to-subcutaneous fat area (VFA/SFA) ratio and RC from fully adjusted linear regression models within each subgroup. P for interaction was obtained by adding a product term between VFA/SFA and the stratifying variable to the model.

All models were adjusted for age, sex, HbA1c, eGFR, systolic blood pressure, current smoking and current drinking, except for the stratifying variable itself.

For HbA1c-stratified analyses, only patients with available HbA1c data were included (n=671).

Abbreviations as in **Table 1**.

## 4 Discussion

In this cross-sectional study of adults with T2D, we systematically compared general and central adiposity indices in relation to RC. We found that indices reflecting central and visceral adiposity, such as waist circumference, waist-to-height ratio (WHtR), VFA and the VFA-to-subcutaneous fat area ratio (VFA/SFA), showed stronger and more consistent associations with RC than general measures such as body weight and BMI, particularly after multivariable adjustment. For key central adiposity indices, we also observed non-linear dose–response patterns, with RC levels rising more steeply beyond certain ranges of increased abdominal or visceral fat. Taken together, these findings extend the concept of residual cardiovascular risk in T2D by linking RC more closely to the burden of visceral adiposity, and support the notion that central obesity may act as an important upstream driver of remnant lipoprotein accumulation and RC elevation beyond LDL-C–centered risk factors.

Several clinical and epidemiological studies have highlighted that visceral and abdominal adiposity are more strongly linked to adverse lipid profiles and CVD than general obesity defined by BMI (30). Imaging-based studies using computed tomography have shown that greater visceral adiposity is associated with a typical atherogenic lipid pattern, characterized by elevated triglycerides, low HDL-C, and increased small, dense low-density lipoproteins (31). Moreover, visceral fat predicts coronary events more robustly than body mass index or total fat mass (32). Similar associations have been observed when visceral adiposity is assessed using composite indices, such as the visceral adiposity index or computed tomography–derived visceral fat area (33). These measures correlate positively with triglycerides, non–HDL-C, and other markers of lipid metabolism disturbance (34).

More recently, several studies have directly examined the relationship between visceral fat and remnant cholesterol (35). Cross-sectional analyses in both general and high-risk populations show that a greater visceral adipose tissue area and visceral fat–related ratios are associated with higher remnant cholesterol levels and an increased burden of remnant lipoproteins (36). In contrast, subcutaneous fat and total body fat show weak or no associations with remnant cholesterol. These findings support the view that remnant cholesterol is more closely linked to ectopic and visceral fat accumulation than to simple expansion of fat mass (37).

Systematic reviews and meta-analyses consistently show that simple measures of central obesity can outperform body mass index as screening tools for cardiometabolic risk (38). Waist-to-height ratio is the most consistent example. In several analyses, it also performs better than waist circumference. These outcomes include incident diabetes, cardiovascular disease, and cardiovascular mortality (39). The advantage is particularly evident in Asian populations. In adults with type 2 diabetes, our findings align with this literature. Indices reflecting visceral and abdominal adiposity show stronger associations with remnant cholesterol than body mass index or body weight (29).

In our analyses, visceral fat area, the visceral-to-subcutaneous fat ratio, and waist-to-height ratio were more informative for remnant cholesterol than general adiposity indices. This supports central obesity as a key clinical phenotype linking excess adiposity to remnant cholesterol–related residual cardiovascular risk (40).

Several biological mechanisms may underlie the stronger associations between visceral adiposity and RC observed in our study and in previous work. Visceral adipose tissue has higher lipolytic activity than subcutaneous fat and releases more free fatty acids (FFAs) into the portal circulation (41). The increased FFA flux to the liver stimulates hepatic very-low-density lipoprotein (VLDL) production, promoting hypertriglyceridaemia and the accumulation of triglyceride-rich lipoproteins and their remnants, thereby raising RC levels (42). In parallel, visceral obesity is typically accompanied by insulin resistance and chronic low-grade inflammation, which can impair the clearance of remnant lipoproteins by altering lipoprotein lipase activity and hepatic receptor-mediated uptake (43). A higher VFA-to-subcutaneous fat area ratio (VFA/SFA) may further indicate a relative loss of “buffering” capacity in subcutaneous depots and a shift towards more ectopic and visceral fat deposition, amplifying these adverse effects on remnant lipoprotein metabolism (44). Together, these pathways provide a plausible biological link between central obesity, disturbances in remnant lipoprotein handling and RC elevation in adults with type 2 diabetes.

Notably, our baseline data revealed a paradoxical trend where LDL-C levels were significantly lower in the highest RC quartile compared with the lowest quartile. This finding suggests that individuals with a higher burden of visceral adiposity and RC may have undergone more intensive lipid-lowering therapy in clinical practice, likely due to their perceived higher cardiovascular risk. The persistence of elevated RC despite relatively lower LDL-C levels in these patients underscores the fact that RC represents a robust component of residual risk that is not fully captured or mitigated by current LDL-C-targeted management.

From a clinical perspective, our findings suggest that relying solely on general measures such as BMI or body weight may underestimate RC–related risk in adults with type 2 diabetes. Indices that capture central and visceral adiposity appear more informative for identifying patients with a higher remnant cholesterol burden. Key examples include visceral fat area and the visceral-to-subcutaneous fat ratio (45). When these measures are unavailable, waist circumference or waist-to-height ratio can be used. Incorporating these indices into routine assessment may help clinicians recognize a subgroup of patients with persistently elevated remnant cholesterol despite management focused on LDL cholesterol. These patients may benefit from more intensive lifestyle modification, weight reduction, and optimization of lipid lowering therapy (46). Where direct measurement of visceral fat is feasible, visceral fat area or the visceral to subcutaneous fat ratio may refine risk stratification and help monitor the effects of interventions aimed at reducing visceral fat (47). Overall, our results support a more phenotype oriented approach to obesity management in type 2 diabetes, with specific attention to central obesity as a modifiable driver of remnant lipoprotein burden and residual cardiovascular risk (48). It is worth noting that while imaging techniques like CT or MRI are considered gold standards for adiposity quantification, bioelectrical impedance analysis (BIA) provides a practical and reliable alternative in clinical settings (49). In the context of the MMC, the use of professional-grade BIA is particularly valuable due to its non-invasive nature, lack of radiation, and cost-effectiveness, allowing for rapid and repeatable assessments of VFA and SFA during routine outpatient visits. The high accessibility of BIA facilitates the identification of ‘at-risk’ individuals with visceral obesity who might otherwise be overlooked by BMI alone, thereby supporting personalized risk stratification and the monitoring of long-term metabolic health in patients with type 2 diabetes. Our findings also highlight the need for prospective and interventional studies to determine whether reducing visceral adiposity can lower remnant cholesterol and improve cardiovascular outcomes (50).

This study has several strengths. We used data from a real-world Metabolic Management Centre cohort of adults with type 2 diabetes and systematically compared a broad panel of general and central adiposity indices—including body weight, BMI, waist circumference, waist-to-height ratio, VFA, subcutaneous fat area (SFA) and the VFA/SFA ratio—in relation to RC. However, several limitations should be acknowledged. First, the single-center, cross-sectional design in Chinese adults with type 2 diabetes limits our ability to establish causal relationships and may affect the generalizability of the findings to other ethnic groups or regions. Second, the reduction in sample size (from 1,135 to 941 participants) due to missing covariates for the fully adjusted models may have introduced a degree of selection bias. Third, while BIA is a practical tool for clinical screening, its quantitative precision for estimating VFA and SFA is lower than that of imaging ‘gold standards’ such as CT or MRI, which may lead to a degree of measurement error. Fourth, the retrospective nature of this study resulted in missing data on lifestyle factors, such as diet and exercise, as well as granular details on lipid-lowering therapies (e.g., specific statin types and dosages). Consequently, the potential for residual confounding cannot be entirely ruled out. Finally, as our analysis focused on RC as an intermediate lipid phenotype without longitudinal follow-up for clinical endpoints and lacked external validation using independent databases, the prognostic relevance and generalizability of these findings warrant further validation in prospective and multi-center cohorts.

## 5 Conclusion

In adults with type 2 diabetes, central and visceral adiposity indices—especially VFA, the VFA/SFA ratio and WHtR—were more strongly associated with residual cholesterol than body weight or BMI. Central obesity may therefore be a key clinical marker of RC-related residual cardiovascular risk, warranting further prospective evaluation.

## Data Availability

The original contributions presented in the study are included in the article/supplementary material. Further inquiries can be directed to the corresponding authors.

## References

[1] Rawshani A Rawshani A Franzén S Sattar N Eliasson B Svensson A . Risk factors, mortality, and cardiovascular outcomes in patients with type 2 diabetes. N Engl J Med. (2018) 379:633. doi: 10.1056/nejmoa1800256. PMID: 30110583

[2] Pennells L Kaptoge S Østergaard H Read S Carinci F Franch-Nadal J . SCORE2-diabetes: 10-year cardiovascular risk estimation in type 2 diabetes in Europe. Eur Heart J. (2023) 44:2544–56. doi: 10.1093/eurheartj/ehad260. PMID: 37247330 PMC10361012

[3] Fu L Tai S Sun J Zhang N Zhou Y Xing Z . Remnant cholesterol and its visit-to-visit variability predict cardiovascular outcomes in patients with type 2 diabetes: Findings from the ACCORD cohort. Diabetes Care. (2022) 45:2136–43. doi: 10.2337/dc21-2511. PMID: 35834242 PMC9472497

[4] Wan E Fung C Yu E Chin W Fong D Chan A . Effect of multifactorial treatment targets and relative importance of hemoglobin A1c, blood pressure, and low‐density lipoprotein‐cholesterol on cardiovascular diseases in Chinese primary care patients with type 2 diabetes mellitus: A population‐based retrospective cohort study. J Am Heart Assoc: Cardiovasc Cerebrovascular Dis. (2017) 6(8):e006400. doi: 10.1161/jaha.117.006400. PMID: 28862945 PMC5586469

[5] Chait A Ginsberg H Vaisar T Heinecke J Goldberg I Bornfeldt K . Remnants of the triglyceride-rich lipoproteins, diabetes, and cardiovascular disease. Diabetes. (2020) 69:508–16. doi: 10.2337/dbi19-0007. PMID: 32198194 PMC7085249

[6] Langsted A Madsen C Nordestgaard B . Contribution of remnant cholesterol to cardiovascular risk. J Internal Med. (2020) 288:116–27. doi: 10.1111/joim.13059. PMID: 32181933

[7] Wadström B Pedersen K Wulff A Nordestgaard B . Elevated remnant cholesterol, plasma triglycerides, and cardiovascular and non-cardiovascular mortality. Eur Heart J. (2023) 44(16):1432–1445. doi: 10.1093/eurheartj/ehac822 36631967

[8] Kristensen F Christensen D Mortensen M Maeng M Kahlert J Sørensen H . Triglycerides and risk of cardiovascular events in statin-treated patients with newly diagnosed type 2 diabetes: a Danish cohort study. Cardiovasc Diabetol. (2023) 22(1):187. doi: 10.1186/s12933-023-01921-5. PMID: 37495999 PMC10373341

[9] Hu X Liu Q Guo X Wang W Yu B Liang B . The role of remnant cholesterol beyond low-density lipoprotein cholesterol in diabetes mellitus. Cardiovasc Diabetol. (2022) 21(1):117. doi: 10.1186/s12933-022-01554-0. PMID: 35761281 PMC9238255

[10] Huh J Roh E Lee S-J Ihm S Han K Kang J . Remnant cholesterol is an independent predictor of type 2 diabetes: A nationwide population-based cohort study. Diabetes Care. (2022) 46(2):305–312. doi: 10.2337/dc22-1550. PMID: 36469354

[11] Drucker D . Prevention of cardiorenal complications in people with type 2 diabetes and obesity. Cell Metab. (2024) 36(2):338–353. doi: 10.1016/j.cmet.2023.12.018. PMID: 38198966

[12] Koenen M Hill M Cohen P Sowers J . Obesity, adipose tissue and vascular dysfunction. Circ Res. (2021) 128:951–68. doi: 10.1161/circresaha.121.318093. PMID: 33793327 PMC8026272

[13] Jayedi A Soltani S Motlagh S Emadi A Shahinfar H Moosavi H . Anthropometric and adiposity indicators and risk of type 2 diabetes: systematic review and dose-response meta-analysis of cohort studies. BMJ. (2022) 376:e067516. doi: 10.1136/bmj-2021-067516. PMID: 35042741 PMC8764578

[14] Fox C Massaro J Hoffmann U Pou K Maurovich-Horvát P Liu C . Abdominal visceral and subcutaneous adipose tissue compartments: Association with metabolic risk factors in the Framingham Heart Study. Circulation. (2007) 116:39–48. doi: 10.1161/CIRCULATIONAHA.106.675355 17576866

[15] Liu J Fox C Hickson D May W Hairston K Carr J . Impact of abdominal visceral and subcutaneous adipose tissue on cardiometabolic risk factors: the Jackson Heart Study. J Clin Endocrinol Metab. (2010) 95:5419–26. doi: 10.1210/jc.2010-1378. PMID: 20843952 PMC2999970

[16] Tchernof A Despres J . Pathophysiology of human visceral obesity: an update. Physiol Rev. (2013) 93:359–404. doi: 10.1152/physrev.00033.2011. PMID: 23303913

[17] Sweatt K Garvey W Martins C . Strengths and limitations of BMI in the diagnosis of obesity: What is the path forward? Curr Obes Rep. (2024) 13:584–95. doi: 10.1007/s13679-024-00580-1. PMID: 38958869 PMC11306271

[18] Borruel S Moltó J Alpañés M Fernández-Durán E Álvarez-Blasco F Luque‐Ramírez M . Surrogate markers of visceral adiposity in young adults: Waist circumference and body mass index are more accurate than waist hip ratio, model of adipose distribution and visceral adiposity index. PloS One. (2014) 9(12):e114112. doi: 10.1371/journal.pone.0114112. PMID: 25479351 PMC4257592

[19] Qiao T Luo T Pei H Yimingniyazi B Aili D Aimudula A . Association between abdominal obesity indices and risk of cardiovascular events in Chinese populations with type 2 diabetes: a prospective cohort study. Cardiovasc Diabetol. (2022) 21(1):225. doi: 10.1186/s12933-022-01670-x. PMID: 36320060 PMC9628026

[20] Yang L Ge Y Zhu Q-Y Zhang Q Wang L Wang X . The combination of fat distribution and BMI redefines obesity: Result from NHANES. J Cachexia Sarcopenia Muscle. (2025) 16(4):e70013. doi: 10.1002/jcsm.70013. PMID: 40662213 PMC12260474

[21] Klein H Zelicha H Meir Y Rinott E Tsaban G Kaplan A . Visceral adipose tissue area and proportion provide distinct reflections of cardiometabolic outcomes in weight loss; pooled analysis of MRI-assessed CENTRAL and DIRECT PLUS dietary randomized controlled trials. BMC Med. (2025) 23(1):57. doi: 10.1186/s12916-025-03891-9. PMID: 39901232 PMC11792534

[22] Kaess B Pedley A Massaro J Murabito J Hoffmann U Fox C . The ratio of visceral to subcutaneous fat, a metric of body fat distribution, is a unique correlate of cardiometabolic risk. Diabetologia. (2012) 55:2622–30. doi: 10.1007/s00125-012-2639-5. PMID: 22898763 PMC3636065

[23] Browning L Hsieh S Ashwell M . A systematic review of waist-to-height ratio as a screening tool for the prediction of cardiovascular disease and diabetes: 0·5 could be a suitable global boundary value. Nutr Res Rev. (2010) 23:247–69. doi: 10.1017/s0954422410000144. PMID: 20819243

[24] Jakubiak G Badicu G Surma S Waluga-Kozłowska E Chwalba A Pawlas N . The visceral adiposity index and its usefulness in the prediction of cardiometabolic disorders. Nutrients. (2025) 17(14):2374. doi: 10.3390/nu17142374. PMID: 40732999 PMC12298961

[25] Abraham A Cule M Thanaj M Basty N Hashemloo M Sorokin E . Genetic evidence for distinct biological mechanisms that link adiposity to type 2 diabetes: Toward precision medicine. Diabetes. (2024) 73:1012–25. doi: 10.2337/db23-1005. PMID: 38530928 PMC11109787

[26] Wang D Chen Z Wu Y Ren J Shen D Hu G . Association between two novel anthropometric measures and type 2 diabetes in a Chinese population. Diabetes. (2024) 26:3238–47. doi: 10.1111/dom.15651. PMID: 38783824

[27] Ding L Fan Y He J Wang R He Q Cui J . Different indicators of adiposity and fat distribution and cardiometabolic risk factors in patients with type 2 diabetes. Obesity. (2021) 29(5):837–845. doi: 10.1002/oby.23151. PMID: 33899339 PMC9115840

[28] Assani M-Z Novac M Dijmărescu A Vaduva C Vladu I Clenciu D . Potential association between atherogenic coefficient, prognostic nutritional index, and various obesity indices in diabetic nephropathy. Nutrients. (2025) 17(8):1339. doi: 10.3390/nu17081339. PMID: 40284203 PMC12030341

[29] Dhokte S Czaja K . Visceral adipose tissue: The hidden culprit for type 2 diabetes. Nutrients. (2024) 16(7):1015. doi: 10.3390/nu16071015. PMID: 38613048 PMC11013274

[30] Powell-Wiley T Poirier P Burke L Despres J Gordon-Larsen P Lavie C . Obesity and cardiovascular disease: A scientific statement from the American Heart Association. Circulation. (2021) 143(21):e984–e1010. doi: 10.1161/cir.0000000000000973. PMID: 33882682 PMC8493650

[31] Hwang Y Fujimoto W Hayashi T Kahn S Leonetti D Boyko E . Increased visceral adipose tissue is an independent predictor for future development of atherogenic dyslipidemia. J Clin Endocrinol Metab. (2016) 101:678–85. doi: 10.1210/jc.2015-3246. PMID: 26636177 PMC4880131

[32] Agrawal S Klarqvist M Diamant N Stanley T Ellinor P Mehta N . BMI-adjusted adipose tissue volumes exhibit depot-specific and divergent associations with cardiometabolic diseases. Nat Commun. (2023) 14(1):266. doi: 10.1038/s41467-022-35704-5. PMID: 36650173 PMC9844175

[33] Xu W Xu C Liu F Chen Y Shi J . Diagnostic and prognostic value of the Chinese visceral adipose index in patients with coronary heart disease and metabolic syndrome undergoing percutaneous coronary intervention: a single-centre retrospective study. BMJ Open. (2025) 15(11):e104778. doi: 10.1136/bmjopen-2025-104778. PMID: 41263916 PMC12636992

[34] Islam MS Wei P Suzauddula M Nime I Feroz F Acharjee M . The interplay of factors in metabolic syndrome: understanding its roots and complexity. Mol Med. (2024) 30(1):279. doi: 10.1186/s10020-024-01019-y. PMID: 39731011 PMC11673706

[35] Skudder-Hill L Sequeira-Bisson I Ko J Cho J Poppitt S Petrov M . Remnant cholesterol, but not low‐density lipoprotein cholesterol, is associated with intra‐pancreatic fat deposition. Diabetes. (2023) 25:3337–46. doi: 10.1111/dom.15233. PMID: 37529874

[36] Luo Y Xiaojing Shen Y Hao Y-P Hu Y Xiao Y . Positive relationship between serum low-density lipoprotein cholesterol levels and visceral fat in a Chinese nondiabetic population. PloS One. (2014) 9(11):e112715. doi: 10.1371/journal.pone.0112715. PMID: 25398089 PMC4232522

[37] Lee M-J Kim J . The pathophysiology of visceral adipose tissues in cardiometabolic diseases. Biochem Pharmacol. (2024) 222:116116. doi: 10.1016/j.bcp.2024.116116. PMID: 38460909 PMC11407912

[38] Xia X Chen S Tian X Xu Q Zhang Y Zhang X . Roles of general and central adiposity in cardiometabolic multimorbidity: revisiting the obesity paradox using a multistate model. Obesity. (2024) 32:810–21. doi: 10.1002/oby.23980. PMID: 38282432

[39] Ashwell M Gunn P Gibson S . Waist‐to‐height ratio is a better screening tool than waist circumference and BMI for adult cardiometabolic risk factors: systematic review and meta‐analysis. Obes Rev. (2012) 13(3):275–86. doi: 10.1111/j.1467-789x.2011.00952.x. PMID: 22106927

[40] Varbo A Freiberg J Nordestgaard B . Remnant cholesterol and myocardial infarction in normal weight, overweight, and obese individuals from the Copenhagen General Population Study. Clin Chem. (2018) 64:219–30. doi: 10.1373/clinchem.2017.279463. PMID: 29021326

[41] Hannukainen J Kalliokoski K Borra R Viljanen A Janatuinen T Kujala U . Higher free fatty acid uptake in visceral than in abdominal subcutaneous fat tissue in men. Obesity. (2010) 18(2):261–5. doi: 10.1038/oby.2009.267. PMID: 19696757

[42] Heeren J Scheja L . Metabolic-associated fatty liver disease and lipoprotein metabolism. Mol Metab. (2021) 50:101238. doi: 10.1016/j.molmet.2021.101238. PMID: 33892169 PMC8324684

[43] Chan D Watts G Barrett P Mamo J Redgrave T . Markers of triglyceride-rich lipoprotein remnant metabolism in visceral obesity. Clin Chem. (2002) 48:278–83. doi: 10.1093/clinchem/48.2.278 11805008

[44] Zhang D Li L-P Zhang Y-H Wang P Lei L Hu Y . Magnetic resonance imaging quantifies causal body compartment effects on metabolic traits via immune phenotypes—a Mendelian randomisation study. J Transl Med. (2025) 23(1):1158. doi: 10.1186/s12967-025-07241-4. PMID: 41126336 PMC12541995

[45] Von Krüchten R Lorbeer R Müller-Peltzer K Rospleszcz S Storz C Askani E . Association between adipose tissue depots and dyslipidemia: The KORA-MRI population-based study. Nutrients. (2022) 14(4):797. doi: 10.3390/nu14040797. PMID: 35215449 PMC8879798

[46] Chu J Zhang H Wu Y Huang Y Zhu T Zhou Z . Efficacy of lifestyle modification combined with GLP-1 receptor agonists on body weight and cardiometabolic biomarkers in individuals with overweight or obesity: a systematic review and meta-analysis. eClinicalMedicine. (2025) 88:103464. doi: 10.1016/j.eclinm.2025.103464. PMID: 40926900 PMC12414836

[47] Emamat H Jamshidi A Farhadi A Ghalandari H Ghasemi M Tangestani H . The association between the visceral to subcutaneous abdominal fat ratio and the risk of cardiovascular diseases: a systematic review. BMC Public Health. (2024) 24(1):1827. doi: 10.1186/s12889-024-19358-0. PMID: 38982435 PMC11232263

[48] Muijsenberg A Canfora E Blaak E . Metabolic phenotypes, genotypes, and gut microbiome signatures in obesity: Implications for precision nutrition strategies in type 2 diabetes prevention. Nutr Rev. (2025) 84(1):158–188. doi: 10.1093/nutrit/nuaf088. PMID: 40587382 PMC12696375

[49] Xu Z Liu Y-O Yan C Yang R Xu L Guo Z . Measurement of visceral fat and abdominal obesity by single-frequency bioelectrical impedance and CT: a cross-sectional study. BMJ Open. (2021) 11(10):e048221. doi: 10.1136/bmjopen-2020-048221. PMID: 34635516 PMC8506854

[50] Bays H Kirkpatrick C Maki K Toth P Morgan R Tondt J . Obesity, dyslipidemia, and cardiovascular disease: A joint expert review from the Obesity Medicine Association and the National Lipid Association 2024. J Clin Lipidol. (2024) 18(3):e320–e350. doi: 10.1016/j.obpill.2024.100108. PMID: 38664184

